# Supplementary figures and images for: Variation in genome content and predatory phenotypes between Bdellovibrio sp. NC01 isolated from soil and B. bacteriovorus type strain HD100
Source: Microbiology (Reading). 2019 Oct 8;165(12):1315–30. doi: 10.1099/mic.0.000861 (PMC7137782; doi:10.1099/mic.0.000861)

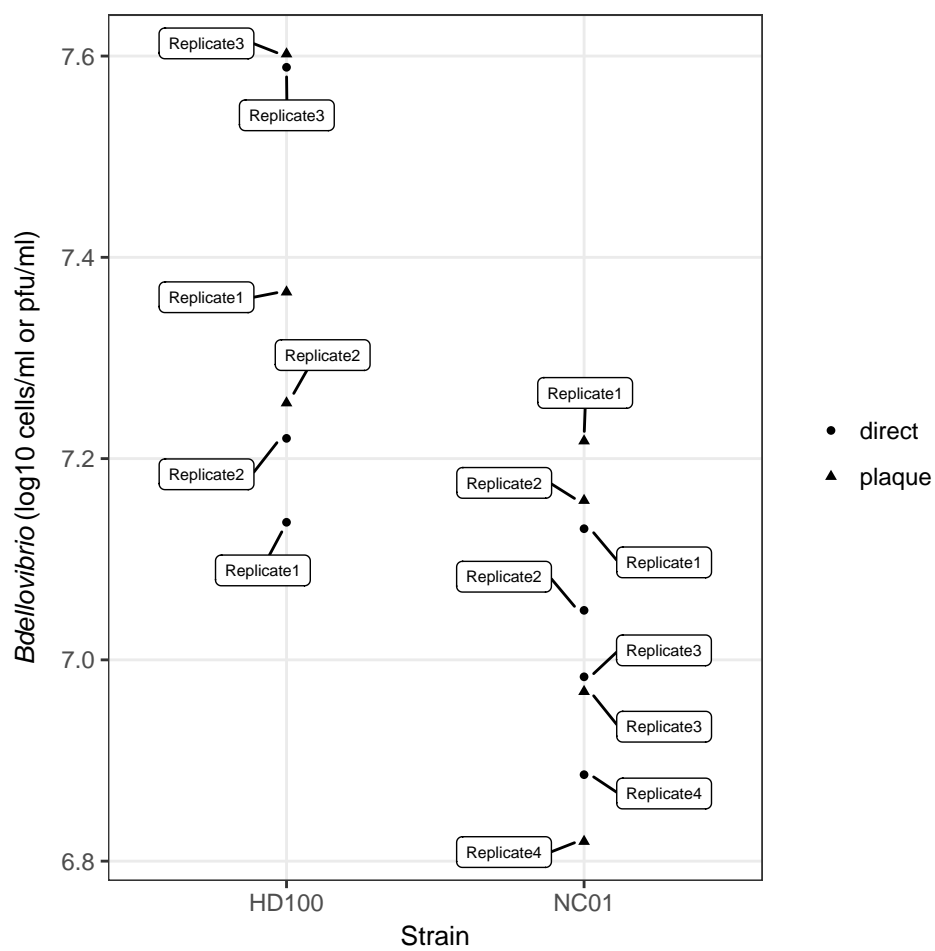

Supplement: Supplementary File 2 [file mic-165-1315-s002.pdf]
